# A multi-model fusion approach incorporating conventional radiological and machine learning features across age spectrum for periorbital fat status prediction

**DOI:** 10.3389/fmed.2026.1752016

**Published:** 2026-02-25

**Authors:** Meng Wang, Yudi Han, Li Li, Xi Lu, Yiqing Jia, Lingli Guo, Yan Han

**Affiliations:** 1 Department of Plastic and Reconstructive Surgery, The First Medical Center, Chinese PLA General Hospital, Beijing, China; 2 Central Medical Branch of PLA General Hospital, Beijing, China; 3 Department of Emergency Medicine, The Sixth Medical Center, Chinese PLA General Hospital, Beijing, China

**Keywords:** machine learning, MRI, periorbital fat, radiomics, stacking ensemble learning

## Abstract

**Objectives:**

To develop an ensemble learning model fusing conventional radiomics (CR) and machine learning (ML) features to assess periorbital fat status across the entire age spectrum.

**Methods:**

Retrospective analysis was conducted on preoperative cranial and facial MRI data of meningioma patients. Patients were categorized into youth, middle-aged, and senior groups and allocated to training and test sets through stratified random sampling. CR and ML features of fat in three periorbital regions were extracted to develop an ensemble learning model, with its clinical application value subsequently evaluated.

**Results:**

237 patients were enrolled: 165 in the training set and 72 in the test set. The training set comprised 19 youth cases (28.5 ± 5.0, 7 male), 41 middle-aged cases (42.9 ± 4.7, 9 male), and 105 senior cases (60.0 ± 6.5, 26 male). The test set included 8 youth cases (28.6 ± 5.6, 4 male), 18 middle-aged cases (43.9 ± 4.1, 6 male), and 46 senior cases (58.8 ± 6.7, 10 male). The ensemble learning model outperformed the CR model, the ML model, and the CR-ML fusion model on the test set, achieving an AUC-macro of 0.833 (95% CI: 0.737–0.902), an F1-score of 0.614, an accuracy (Acc) of 0.597, and a positive predictive value (PPV) of 0.690. Ensemble learning models demonstrated optimal comprehensive capabilities in multi-classification tasks, enhancing generalization and robustness.

**Conclusion:**

Our ensemble learning model achieved non-invasive and reliable assessment of periorbital fat status across the entire age spectrum, enriching the evaluation methodology for rejuvenation surgery.

## 1 Introduction

The eyes and periorbital areas, as central components of the midface region, represent the visual focus of the face and are one of the primary targets for facial rejuvenation surgery. With advancing age, the periorbital tissues—including skin, fascia, fat, muscle, and bone—undergo varying degrees of alteration across the entire age spectrum (1–3). In the midface, aging characteristics predominantly correlate with soft tissue changes (4). Consequently, comprehensively understanding the trajectory of age-related soft tissue changes guides clinicians performing periorbital rejuvenation procedures. For instance, it enables precise preoperative prediction of treatment strategies tailored to specific age groups, such as determining the volume of periorbital fat to be excised or grafted, or planning localized injections of fillers and nutraceuticals (5–7). Previous studies on periorbital fat morphology are predominantly based on cadaveric dissection (8, 9). This methodology, however, is constrained by limited sample sizes and postmortem tissue alterations, and thus fails to reflect accurately the characteristics of living tissues.

Imaging data of the mid facial region (CT, MRI) represent clinical data reflecting the true status of various tissues (10, 11). However, they do not permit direct assessment of tissue conditions due to the absence of quantitative data and intuitive features. In recent years, numerous researchers have attempted to quantify facial characteristics to indirectly evaluate changes in deep tissues. Examples include: using 3D facial photography to measure periorbital volume changes as a proxy for periorbital fat volume alterations (12, 13); applying scoring systems to assess periorbital tissue status (14); and utilizing grayscale values from periorbital photographs to evaluate fat grafting efficacy (15). Studies have also employed tomographic imaging (CT, MRI) data to investigate relationships between periorbital or facial fat and aging. Nevertheless, these studies rely on overly simplistic and limited metrics—such as thickness at different anatomical levels, maximum width, volume, and positional changes of periorbital fat (16–18). While such research offers preliminary insights into age-related periorbital fat dynamics, comprehensive imaging studies across the full age spectrum with large sample sizes and intelligent analytical approaches remain rarely explored.

Development of a foundational age-prediction model required extraction of conventional radiomics (CR) and machine learning (ML) features from periorbital fat on MRI scans across the entire age spectrum. We enhanced the model’s comprehensive capability through feature fusion and ensemble learning methodologies. As a preliminary exploratory investigation, this research primarily aimed to establish a clinical prediction model fusing conventional imaging and ML techniques. The objective was to improve clinicians’ assessment proficiency regarding periorbital fat status in patients of various age groups, thereby providing guidance for periocular rejuvenation therapies.

## 2 Materials and methods

### 2.1 Patient population and selection

The study obtained approval from the ethics committee of the First Medical Center of Chinese PLA General Hospital (No. 2025–071). As a single-center retrospective study, it waived the requirement for informed consent forms.

**Figure 1:**
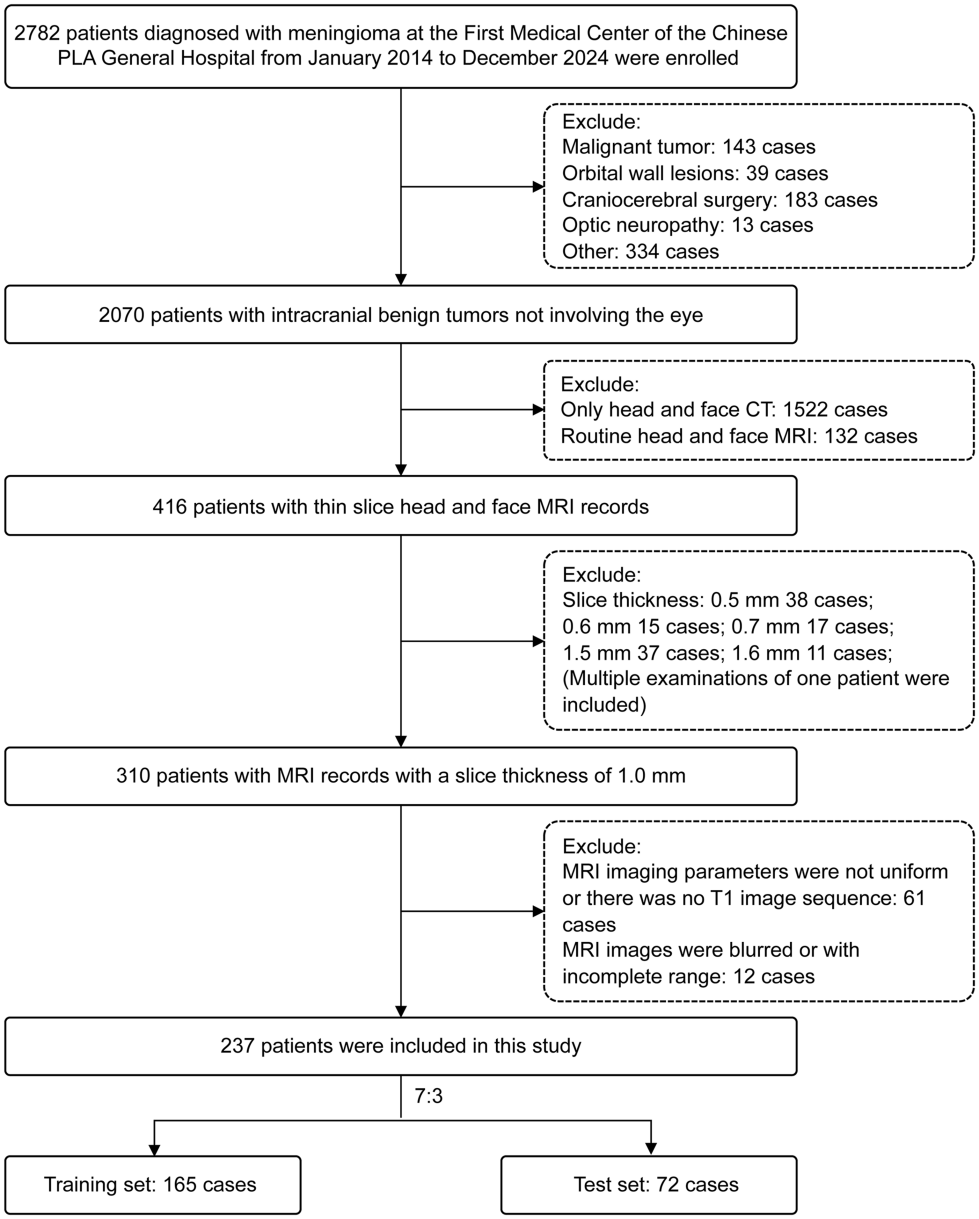
The flow chart for patient screening.

Imaging data were retrospectively collected from patients who underwent cranial and facial MRI scans for meningioma at the First Medical Center of Chinese PLA General Hospital between January 2014 and December 2024. All cases were stratified by age group (youth group: ≥ 18 and < 35 years; middle-aged group: ≥ 35 and < 60 years; senior group: ≥ 60 years) and randomly divided into training and test sets in a 7:3 ratio using stratified random sampling. A total of 237 patients were included (Figure 1), with 27 in the youth group, 59 in the middle-aged group, and 151 in the senior group (Table 1, Table 2).

**Table 1:** Baseline characteristics of patients in each group.

Category	Youth group (*n* = 27)		Middle-aged group (*n* = 59)		Senior group (*n* = 151)		*p* value
	Female	Male	Female	Male	Female	Male	
Patient	16 (6.8)	11 (4.6)	45 (19.0)	14 (5.9)	115 (48.5)	36 (15.2)	
Age (year)	28.2 ± 5.3	29.1 ± 5.0	43.5 ± 4.7	42.4 ± 3.7	59.5 ± 6.4	59.8 ± 7.2	
BMI (kg/m^2^)	22.8 ± 3.7	25.0 ± 2.6	24.2 ± 2.6	24.6 ± 2.7	24.6 ± 2.4	25.3 ± 1.9	0.3295^#^

Data are shown as number (percentage) or mean ± SD; SD, standard deviation; BMI body mass index. ^#^Analysis results of data differences among various age groups (Kruskal-Wallis H test).

**Table 2:** Baseline characteristics of patients in the training set and test set.

Category	Training set (*n* = 165)	Test set (*n* = 72)	*p* value
Youth group	19	8	
Sex (male)	7 (36.8)	4 (50.0)	
Age (years)	28.5 ± 5.0	28.6 ± 5.6	0.669
BMI (kg/m^2^)	24.4 ± 3.1	22.2 ± 3.7	0.106
Middle-aged group	41	18	
Sex (male)	9 (22.0)	6 (31.6)	
Age (years)	42.9 ± 4.7	43.9 ± 4.1	0.552
BMI (kg/m^2^)	24.2 ± 2.6	24.5 ± 2.6	0.731
Senior group	105	46	
Sex (male)	26 (24.8)	10 (21.7)	
Age (years)	60.0 ± 6.5	58.8 ± 6.7	0.278
BMI (kg/m^2^)	24.8 ± 2.2	24.7 ± 2.5	0.838

Data are shown as number (percentage) or mean ± SD; SD, standard deviation; BMI body mass index. The *t* test is employed to analyze the differences between distinct data sets.

All patients met the following inclusion criteria: (1) age ≥18 years; (2) no history of head or facial surgery, with primary disease excluding periorbital fat involvement; (3) absence of malignant tumors or immune system disorders; (4) no prior radiotherapy, chemotherapy, or glucocorticoid therapy; (5) no history of craniofacial dysplasia; (6) cranial and facial MRI scans with 1-mm slice thickness, covering a range from the skull vertex superiorly to the upper incisor plane inferiorly.

Patients were excluded based on the following criteria: (1) body mass index (BMI) < 18.5 or ≥ 28.0; (2) MRI images with poor resolution or indistinct boundaries, precluding accurate regions of interest (ROI) annotation; (3) incomplete coverage of target regions in cranial and facial MRI scans; (4) absence of T1-weighted sequences in cranial and facial MRI protocols; (5) history of facial or periorbital deformities and prior surgeries.

### 2.2 Collection of clinical information and MRI images

Clinical information and MRI images were collected retrospectively from the hospital’s electronic medical record system and imaging database. Patients diagnosed with meningioma by the neurosurgery department underwent preoperative high-resolution head and facial T1-weighted imaging, primarily with a slice thickness of 1 mm, meeting the study’s requirements. Identical MRI equipment and unchanging imaging parameters ensured data stability and reliability for this investigation.

### 2.3 MRI acquisition

Preoperative patients routinely underwent cranial and facial MRI scanning using high-resolution T1-weighted imaging. The scanning was performed with the following parameters: slice thickness of 1 mm, interslice gap of 1 mm, matrix size of 260 × 260, and an average of 2 signal acquisitions. The imaging was conducted on a 1.5 T high-field superconducting magnet (Siemens Espree, Erlangen, Germany) equipped with a 32-channel phased-array body coil.

### 2.4 Region of interest segmentation

**Figure 2:**
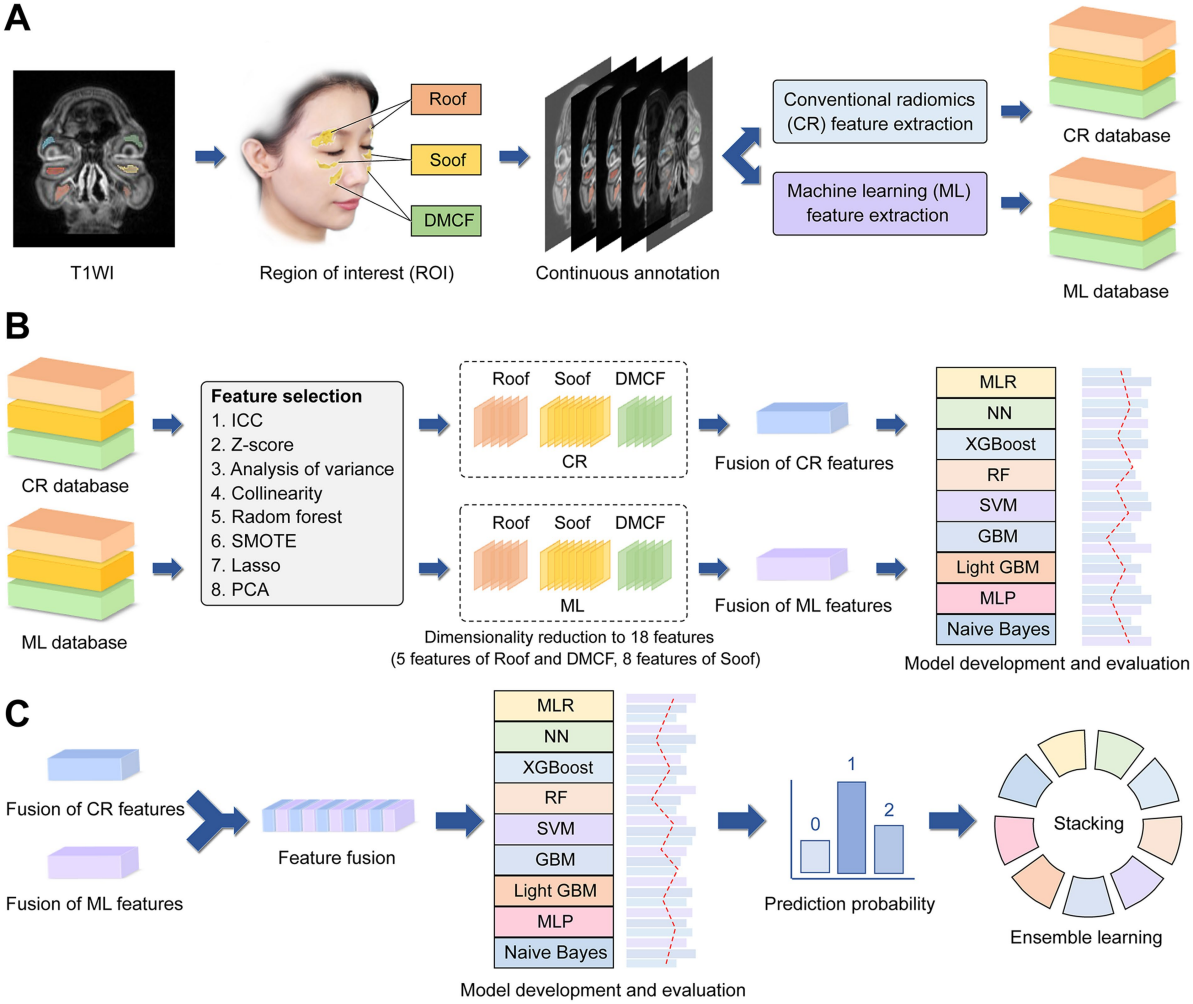
Flowchart of this study which contains three main steps. Extract conventional radiomics (CR) feature data and machine learning (ML) feature data **(A)**. Dimensionality reduction and feature fusion of CR and ML data respectively; development and evaluation of nine distinct models **(B)**. Fusion of CR and ML features; development of a stacking ensemble learning model utilizing the prediction probabilities from the nine models **(C)**.

**Figure 3:**
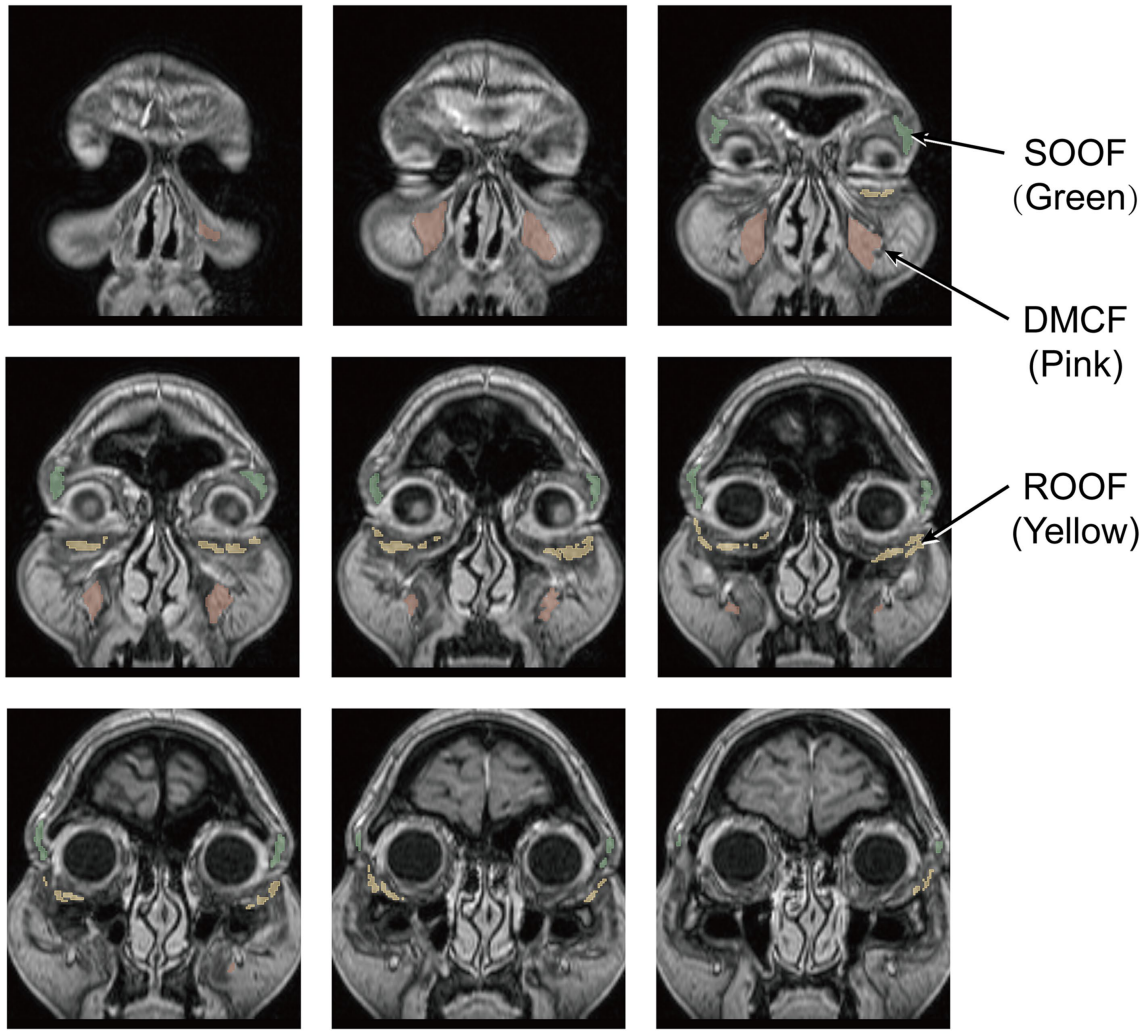
Consecutive coronal T1-weighted images of the face and three ROIs: ROOF (green), SOOF (yellow), and DMCF (pink).

Based on reviewing previous research data (19), three anatomical regions of periorbital fat were identified (Figure 2A, Figure 3): retro-orbicularis oculi fat (ROOF), sub-orbicularis oculi fat (SOOF) and deep medial cheek fat (DMCF). Two senior plastic surgeons (each with over 10 years of clinical experience) independently annotated the ROIs on all imaging data using 3D Slicer software (version 5.7.0). For complex or ambiguous images, annotations were guided by one expert plastic surgeon (with over 20 years of clinical experience). To ensure reproducibility, two senior plastic surgeons concurrently ambiguous imaging data from 50 randomly selected patients; an intraclass correlation coefficient (ICC) evaluation was subsequently performed (20). Metrics with ICC values below the reliability threshold (defined as ICC ≤ 0.75) were excluded as unstable indicators.

### 2.5 Images preprocessing

All craniofacial T1-weighted MRI images underwent N4 bias field correction algorithm using the Python (version v3.9.23) programming language to mitigate potential artifacts arising from local magnetic field in homogeneities. Subsequently, coordinate system standardization was performed on all imaging data to ensure accurate feature extraction.

### 2.6 Conventional radiological features

The open-source library “PyRadiomics” (version v3.1.0) was employed to extract radiomics feature data from segmented ROIs using the Python programming language. Prior to feature extraction, all images were preprocessed: images underwent resampled to a uniform voxel size of 1 × 1 × 1 mm^3^, and grey-level data were discretized into 25 bins using nearest-neighbor interpolation. Ultimately, a total of 1,688 radiomics features were extracted, including 14 shape-based features, 324 first-order statistical features, 432 gray level co-occurrence matrix (GLCM) features, 288 gray level run length matrix (GLRLM) features, 288 gray level size zone matrix (GLSZM) features, 252 gray level dependence matrix (GLDM) features, and 90 neighboring gray tone difference matrix (NGTDM) features.

### 2.7 Machine learning features and model development

Consistent with conventional radiomics, the Python programming language was employed to extract machine learning features from segmented medical images. A 3D ResNet18 backbone network was utilized, optionally embedding squeeze-and-excitation (SE) modules after residual blocks. The SE mechanism compressed spatial information through global average pooling, generated channel-wise weights via fully-connected layers (compression ratio: 16), and recalibrated features using sigmoid activation to enhance discriminative channel responses. Input MRI scans (including images and masks) were uniformly resampled to 1 × 1 × 1 mm^3^ voxels. ROIs were extracted and cropped to minimum bounding boxes, with intensity values normalized to the 0–1 range using min-max scaling. ROI volumes were padded or cropped to 32 × 32 × 32 tensors (zero-padded for undersized volumes), retaining only masked regions. Features were extracted through global average pooling and fully-connected layers, yielding a 512-dimensional output vector without preserved spatial dimensions.

Using CR and ML features extracted from the training set, we conducted separate training procedures for nine different ML models (Figure 2B). Given the sample size and distribution characteristics of the study cohort, five-fold cross-validation with five repeats was employed to enhance model stability during validation. Procedure: (1) Features with ICC > 0.75 were retained due to high stability. All datasets were standardized using the Z-score method. (2) Analysis of variance (ANOVA) screened features exhibiting statistically significant differences (*p* < 0.05) across youth, middle-aged, and senior groups. (3) Pearson and Spearman correlation analyses were applied to normally and non-normally distributed features, respectively, to remove redundant features with correlation coefficients (*r*) > 0.9. (4) Synthetic minority over-sampling technique (SMOTE) was employed to balance sample sizes in the two non-senior subgroups, aligning them with the senior group (*n* = 105). (5) Random forest (RF) feature selection was implemented to retain only features with importance scores meeting or exceeding the mean value. (6) Least absolute shrinkage and selection operator (LASSO) regression was used for feature screening, with the penalty coefficient (*λ*) determined by minimum mean square error (MSE). (7) Principal component analysis (PCA) was synchronously applied to reduce dimensionality of selected features in the training, internal validation, and test sets. (8) nine ML models were trained on the training set, with performance rigorously evaluated on an independent test set.: multiclass logistic regression (MLR), neural network (NN), support vector machine (SVM), multilayer perceptron (MLP), random forest (RF), gradient boosting machine (GBM), light gradient boosting machine (Light GBM), naïve Bayes, and extreme gradient boosting (XGBoost).

### 2.8 Data fusion and stacking ensemble learning models

MRI feature data from each patient were extracted from three ROIs (Figure 2C): Roof, Soof, and DMCF. These regions generated three distinct sets of feature data. Initially, the filtered CR features from these groups were fused through direct concatenation to construct a CR model (identical methodology was applied to the ML model). Subsequently, the screened features from CR and ML were fused through direct concatenation to construct a CR-ML fusion model. Finally, the predicted probabilities generated by the CR-ML fusion model were utilized as training data to build a stacking ensemble learning framework, with a logistic regression classifier employed as the meta-learner.

### 2.9 Statistical analysis

All data analyses were conducted using open-source libraries in Python. The ICC was employed to evaluate feature reproducibility, with ICC > 0.75 indicating good consistency. Model discriminative performance was assessed using the internal validation set and testing set through the following metrics: area under the curve (AUC), AUC macro-average (AUC-macro), 95% confidence interval (CI), accuracy (Acc), positive predictive value (PPV), sensitivity (Sen), F1-score, and confusion matrices. Owing to the imbalanced class distribution in the study sample, AUC-macro (calculated via interpolation) was selected as the primary evaluation metric due to its minimal susceptibility to sample distribution bias and superior stability (21). Based on the macro-average ROC curve, the AUC-macro (Macro_ AUC) is calculated using the trapezoidal rule:


$$\text{Macro}\_\mathrm{AUC}=\sum_{j=1}^{M}(f_{j}-f_{j-1})\cdot \frac{\text{macro}\_\mathrm{tpr}(f_{j})+\text{macro}\_\mathrm{tpr}(f_{j-1})}{2}$$

$f_{j}$ and $f_{j-1}$ are adjacent false positive rate (FPR) grid points. $\text{macro}\_\mathrm{tpr}(f_{j})$ and $\text{macro}\_\mathrm{tpr}(f_{j-1})$ are the macro-average true positive rate (TPR) values at the corresponding FPR points. M is the total number of points in the FPR grid.


$$\text{macro}\_\mathrm{tpr}(f_{j}\;)=\frac{1}{K}\sum_{i=0}^{K}\mathrm{tp}r_{i}(f_{j}\;)$$

K is the total number of classes. $\mathrm{tp}r_{i}(f_{j}\;)$ is the true positive rate for the i-th class at FPR point $f_{j}$ ​, obtained through linear interpolation. $f_{j}$ is the j-th point in the FPR grid, where j = 0, 1, …, M , typically with $f_{0}$ = 0 and $f_{M}$ ​ = 1.

The F1-score, which integrates PPV and Sen, was designated a secondary metric as it may overestimate model performance in imbalanced data; other metrics served as supplementary indicators. To enable precise model comparison, all evaluation results were reported to three decimal places. For normally distributed data with homogeneity of variance, independent samples *t* tests and one-way ANOVA were applied; otherwise, non-parametric tests (Kruskal–Wallis *H* test) were utilized, with statistical significance defined as *p* < 0.05.

## 3 Result

### 3.1 Patient characteristics

The baseline characteristics of the study participants are presented in Tables 1, 2. A total of 237 patients were enrolled: 27 in the young group (11 male), 59 in the middle-aged group (14 male), and 151 in the senior group (36 male). Through stratified sampling, the cohort was divided into a training set (including an internal validation set) of 165 cases: young group (*n* = 19, 28.5 ± 5.0 years, 7 male), middle-aged group (*n* = 41, 42.9 ± 4.7 years, 9 male), and senior group (*n* = 105, 60.0 ± 6.5 years, 26 male); along with a testing set of 72 cases: young group (*n* = 8, 28.6 ± 5.6 years, 4 male), middle-aged group (*n* = 18, 43.9 ± 4.1 years, 6 male), and senior group (*n* = 46, 58.8 ± 6.7 years, 10 male). Within identical age strata, no statistically significant differences in BMI were observed between the training and test sets (*p* > 0.05). Similarly, no statistically significant BMI differences existed across distinct age groups (*p* > 0.05).

### 3.2 Conventional radiological model

Following feature selection and SMOTE oversampling for the Roof, Soof, and DMCF regions respectively, the feature dimensionality of the Roof and DMCF regions was reduced to 5 *via* PCA, while the Soof region was reduced to 8 features. Each patient thus contributed a total of 18 features. All models demonstrated stable performance on the training set (Table 3). In the test set, the Naive Bayes model exhibited optimal results with an AUC-macro of 0.757 (95% CI, 0.628–0.856) and an F1-score of 0.598.

**Table 3:** Predictive performance of CR model, ML model and fusion model (CR-ML).

Model	Training set				Test set			
	Acc (Sen)*	PPV	F1	AUC-macro (95% CI)	Acc (Sen)*	PPV	F1	AUC-macro (95% CI)
MLR								
CR	0.768	0.768	0.767	0.927 (0.902–0.946)	0.583	0.712	0.620	0.748 (0.623–0.843)
ML	0.730	0.729	0.728	0.883 (0.85–0.909)	0.486	0.635	0.504	0.771 (0.651–0.862)
CR-ML	0.819	0.818	0.816	0.943 (0.92–0.96)	0.597	0.721	0.619	0.806 (0.703–0.884)
NN								
CR	0.917	0.92	0.917	0.982 (0.967–0.990)	0.583	0.700	0.614	0.749 (0.639–0.837)
ML	0.895	0.899	0.893	0.980 (0.969–0.987)	0.458	0.666	0.476	0.706 (0.593–0.804)
CR-ML	0.933	0.933	0.933	0.990 (0.980–0.996)	0.597	0.694	0.615	0.819 (0.720–0.893)
XGBoost								
CR	0.813	0.818	0.811	0.929 (0.902–0.946)	0.458	0.642	0.496	0.748 (0.651–0.824)
ML	0.727	0.728	0.720	0.900 (0.87–0.921)	0.417	0.604	0.419	0.721 (0.614–0.807)
CR-ML	0.797	0.801	0.794	0.932 (0.91–0.949)	0.528	0.689	0.548	0.789 (0.692–0.861)
RF								
CR	0.832	0.835	0.829	0.958 (0.941–0.972)	0.528	0.709	0.570	0.727 (0.605–0.822)
ML	0.867	0.870	0.863	0.966 (0.952–0.978)	0.417	0.586	0.422	0.717 (0.619–0.800)
CR-ML	0.902	0.909	0.900	0.977 (0.965–0.987)	0.597	0.725	0.612	0.779 (0.691–0.851)
SVM								
CR	0.933	0.934	0.933	0.99 (0.98–0.995)	0.569	0.652	0.594	0.741 (0.648–0.825)
ML	0.933	0.933	0.933	0.989 (0.979–0.994)	0.500	0.592	0.522	0.727 (0.616–0.817)
CR-ML	0.943	0.946	0.943	0.995 (0.987–0.998)	0.556	0.634	0.579	0.739 (0.637–0.829)
GBM								
CR	0.737	0.737	0.735	0.893 (0.867–0.917)	0.472	0.633	0.508	0.706 (0.583–0.796)
ML	0.775	0.776	0.774	0.901 (0.872–0.925)	0.361	0.502	0.391	0.682 (0.573–0.773)
CR-ML	0.797	0.797	0.796	0.915 (0.889–0.936)	0.500	0.626	0.530	0.716 (0.599–0.816)
Light GBM								
CR	0.775	0.780	0.769	0.925 (0.901–0.944)	0.472	0.669	0.510	0.715 (0.603–0.806)
ML	0.810	0.809	0.805	0.936 (0.914–0.953)	0.403	0.554	0.412	0.676 (0.568–0.773)
CR-ML	0.844	0.854	0.841	0.954 (0.935–0.969)	0.528	0.667	0.561	0.767 (0.668–0.85)
MLP								
CR	0.787	0.785	0.784	0.898 (0.866–0.922)	0.528	0.730	0.579	0.737 (0.634–0.82)
ML	0.730	0.732	0.730	0.877 (0.843–0.906)	0.458	0.589	0.477	0.682 (0.565–0.793)
CR-ML	0.740	0.742	0.737	0.872 (0.834–0.901)	0.542	0.646	0.577	0.713 (0.59–0.818)
Naive bayes								
CR	0.749	0.750	0.747	0.891 (0.858–0.917)	0.556	0.736	0.598	0.757 (0.628–0.856)
ML	0.702	0.699	0.696	0.868 (0.834–0.897)	0.361	0.501	0.365	0.624 (0.506–0.733)
CR-ML	0.778	0.780	0.774	0.911 (0.881–0.934)	0.542	0.732	0.558	0.775 (0.665–0.856)

AUC-macro, AUC macro-average; AUC, the area under the receiver operating characteristic curves; Acc, accuracy; PPV, positive predictive value; Sen, sensitivity; F1, F1-score; CI, confidence interval; CR, conventional radiation; ML, machine learning; MLR, multiclass logistic regression; MLP, multilayer perceptron; NN, convolutional neural network; RF, random forest; GBM, gradient boosting machine; XGBoost, extreme gradient boosting; Light GBM, light gradient boosting machine. *Sen is calculated in multi-class problems using a weighted average, which is mathematically equal to Acc.

### 3.3 ML feature model

Identical to the CR model, each patient contributed a total of 18 ML features. Within the training set evaluation metrics, all models exhibited stable performance, with results which were comparable to those of the CR model (Table 3). In the testing set, the optimal model was MLR, attaining an AUC-macro of 0.771 (95% CI, 0.651–0.862) and an F1-score of 0.504. The predictive capability of models developed using ML features was comparable to that of the CR model; the optimal model in the testing set showed an increase of 0.014 (1.8%) in AUC-macro but exhibited a decrease of 0.095 (−15.8%) in F1-score.

### 3.4 Feature fusion model

To further enhance model performance, we directly concatenated CR and ML feature data to develop a fusion model. In the evaluation results of the CR-ML fusion model (Table 3), the training set metrics remained stable. In the test set, the NN model performed optimally, achieving an AUC-macro of 0.819 (95% CI: 0.720–0.893) and an F1-score of 0.615. Following data fusion, the performance of most models (seven models) improved to varying degrees. Compared to the CR and ML models, the optimal model’s test set AUC-macro increased by 0.062 (8.2%) and 0.048 (6.2%), respectively, and the F1-score increased by 0.017 (2.8%) and 0.111 (22.1%), respectively.

### 3.5 Stacking ensemble learning model

A stacking ensemble learning model was developed using the probability data from both the training and test sets of the CR-ML fusion model, with a logistic regression model selected as the meta-learner. The base models produced nine sets of predicted probability data, and all possible combinations were exhaustively evaluated. As shown in Table 4, the evaluation metrics of the top 10 model combinations were compiled according to their test set AUC-macro ranking. In the training set, all models exhibited excellent performance; in the test set, the stacking model fusing three base models (GMB, Light GBM and NN) achieved optimal performance, with an AUC-macro of 0.833 (95% CI: 0.737–0.902), an F1-score of 0.614, an Acc of 0.597, and a positive predictive value (PPV) of 0.690. Compared to the optimal CR-ML fusion models (Table 5), the top-performing model demonstrated an increase in test set AUC-macro by 0.014 (1.7%), while other metrics remained relatively stable.

**Table 4:** Multi-model stacking ensemble learning evaluation (Top 10).

Model composition	Training set				Test set			
	Acc (Sen)*	PPV	F1	AUC-macro (95% CI)	Acc (Sen)*	PPV	F1	AUC-macro (95% CI)
GMB, Light GBM, NN	0.946	0.946	0.946	0.991 (0.981–0.996)	0.597	0.690	0.614	0.833(0.737–0.902)
Light GBM, NN	0.946	0.946	0.946	0.991(0.981–0.996)	0.597	0.690	0.614	0.833(0.733–0.904)
Light GBM, MLP, NN, Naive bayes	0.946	0.946	0.946	0.990(0.979–0.995)	0.597	0.713	0.617	0.827(0.728–0.900)
GMB, Light GBM, MLP, NN, Naive bayes	0.946	0.946	0.946	0.990(0.978–0.996)	0.597	0.713	0.617	0.826(0.728–0.903)
GMB, Light GBM, MLP, NN, Naive bayes, XGBoost	0.946	0.946	0.946	0.990(0.980–0.996)	0.597	0.713	0.617	0.826(0.725–0.899)
Light GBM, MLP, NN, Naive bayes, XGBoost	0.946	0.946	0.946	0.990(0.979–0.996)	0.597	0.713	0.617	0.826(0.725–0.896)
MLP, NN, Naive bayes	0.943	0.943	0.943	0.989(0.978–0.996)	0.597	0.709	0.616	0.825 (0.725–0.898)
MLP, NN, Naive bayes, XGBoost	0.943	0.943	0.943	0.989(0.978–0.996)	0.597	0.709	0.616	0.825(0.730–0.896)
Light GBM, NN, XGBoost	0.946	0.946	0.946	0.992 (0.983–0.996)	0.597	0.690	0.614	0.825(0.718–0.895)
GMB, MLP, NN, Naive bayes	0.943	0.943	0.943	0.989 (0.979–0.996)	0.597	0.709	0.616	0.825(0.726–0.904)

AUC-macro, AUC macro-average; AUC, the area under the receiver operating characteristic curves; Acc, accuracy; PPV, positive predictive value; Sen, sensitivity; F1, F1-score; CI, confidence interval; CR, conventional radiation; ML, machine learning; MLP, multilayer perceptron; NN, convolutional neural network; GBM, gradient boosting machine; XGBoost, extreme gradient boosting; Light GBM, light gradient boosting machine. *Sen is calculated in multi-class problems using a weighted average, which is mathematically equal to Acc.

**Table 5:** Evaluation metrics of optimal models by method on test set.

Model composition	Acc (sen)*	PPV	F1	AUC-macro (95% CI)
CR (Naive bayes)	0.556	0.736	0.598	0.757 (0.628–0.856)
ML (MLR)	0.486	0.635	0.504	0.771 (0.651–0.862)
CR-ML (NN)	0.597	0.694	0.615	0.819 (0.720–0.893)
Stacking (GMB, Light GBM, NN)	0.597	0.690	0.614	0.833 (0.737–0.902)

AUC-macro, AUC macro-average; AUC, the area under the receiver operating characteristic curves; Acc, accuracy; PPV, positive predictive value; Sen, sensitivity; F1, F1-score; CI, confidence interval; CR, conventional radiation; ML, machine learning; MLR, multiclass logistic regression; NN, convolutional neural network; GBM, gradient boosting machine; Light GBM, light gradient boosting machine. *Sen is calculated in multi-class problems using a weighted average, which is mathematically equal to Acc.

## 4 Discussion

**Figure 4:**
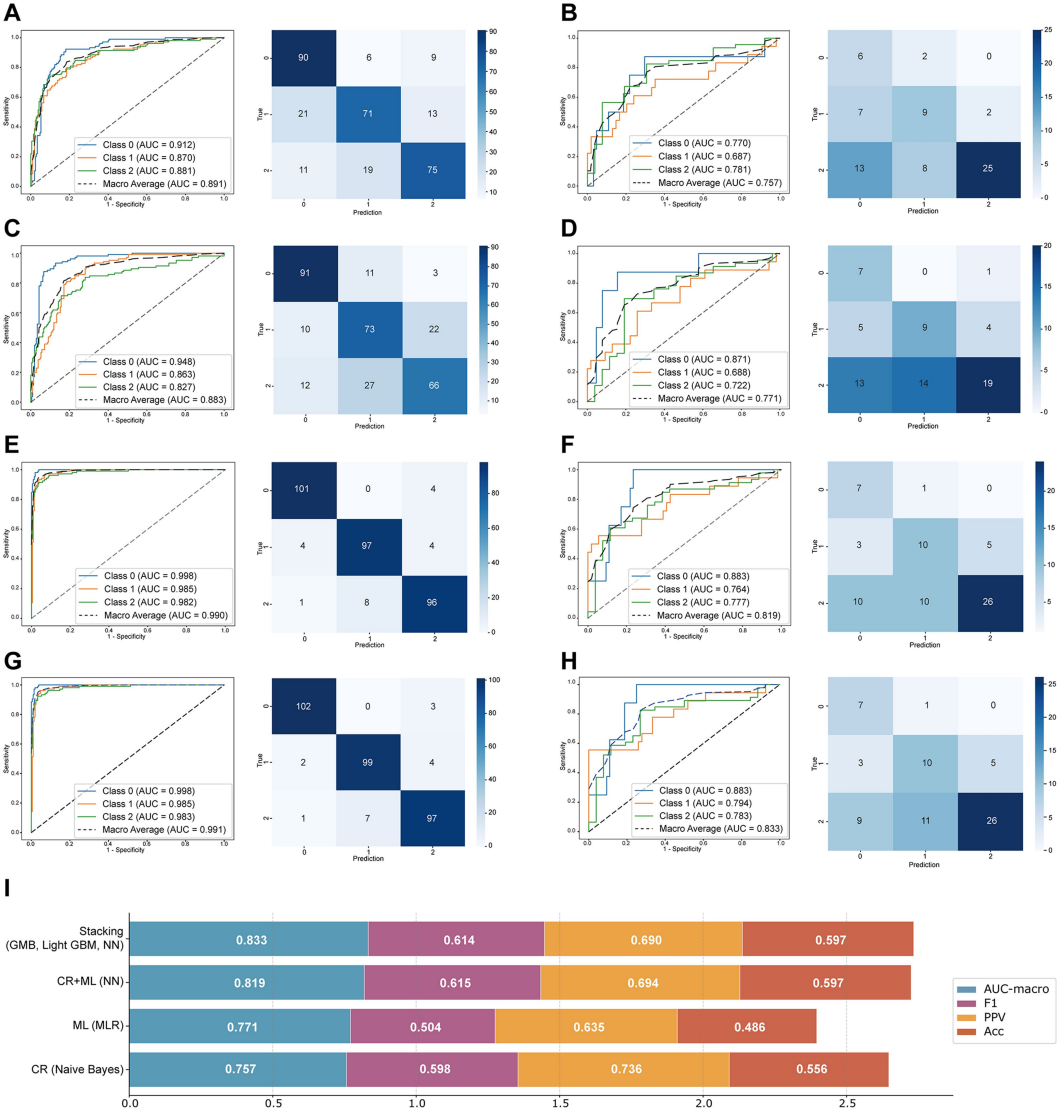
The performance of the optimal model: receiver operating characteristic (ROC) curves and confusion matrices of conventional radiomics (CR) model (Naive Bayes) training set **(A)** and test set **(B)**. ROC curves and confusion matrices of machine learning (ML) model (multiclass logistic regression) training set **(C)** and test set **(D)**. ROC curves and confusion matrices of fusion model (multiclass logistic regression) training set **(E)** and test set **(F)**. ROC curves and confusion matrices of stacking model training set **(G)** and test set **(H)**. Class 0 represents the youth group. Class 1 represents the middle-aged group. Class 2 represents the senior group. Comprehensive evaluation of the optimal models **(I)**.

All models developed independently from CR and ML features alone exhibited limited discriminative capacity, falling below the threshold for reliable clinical deployment (Figures 4A–D). We observed that CR and ML models exhibited complementary strengths in discriminating periorbital fat compartments (different age groups): as shown in Figures 4B,D, both models showed uneven predictive performance across classes in the test set: The CR model outperformed in Class 2 (senior group) with an AUC of 0.781, whereas the ML model excelled in Class 0 (youth group) with an AUC of 0.871. Fusion of these datasets was hypothesized to enhance overall predictive capability. Among CR-ML fusion models, the NN model yielded optimal test-set performance (Figure 4F, Table 3). Key metrics improved significantly: AUC-macro, 0.819 (95% CI: 0.720–0.893); F1-score, 0.615; accuracy, 0.597; PPV, 0.694, collectively demonstrating robust discriminative power. Furthermore, other fusion models exhibited performance gains to varying degrees.

Stacking ensemble learning, as a stacked generalization model, enhanced generalization capability and stability by combining multiple base models, thus improving prediction performance (22). As shown in Table 4, the optimal model combination (GBM, Light GBM, and NN) achieved a test set AUC-macro of 0.833 (0.737–0.902) and an F1-score of 0.614. The AUC values for each class (Class 0, 1, 2) showed improvement (Figures 4G,H), and the predictive capability across all three classes was more balanced, stable, and better suited for the requirements of practical clinical application. From Figure 4I, it is evident that the comprehensive performance of the stacking model surpassed that of the other three models, yielding the highest cumulative values for AUC-macro, F1-score, Acc, and PPV. Further analysis of the magnitude of performance improvement in the stacking model revealed an AUC-macro increase of 0.014 (1.7%) compared to the best CR-ML fusion model (NN). In ML, an AUC improvement >0.01 is typically considered significant (23), while the F1-score, Acc, and PPV remained stable.

The noninvasive, data-driven, and intelligent assessment of periorbital fat status represents a future direction for guiding facial rejuvenation surgery and serves as a critical indicator for evaluating surgical outcomes. Facial fat exhibits regional distribution patterns, with varying degrees of age-related changes across different areas, which is why this study extracted and analyzed features from the three primary periorbital fat compartments—Roof, Soof, and DMCF—separately (24–26). Previous studies indicated that facial fat volume increased with BMI but showed no statistically significant differences based on gender or age (27). Conversely, other research identified age, gender, and BMI as significant factors influencing mid facial fat volume (28). These conclusions, derived from traditional measurement metrics, displayed both consistencies and contradictions, likely due to insufficient data mining of deep-layer fat characteristics—a gap this study aimed to address. Furthermore, all collected cases were of Asian ethnicity (with 97.0% being Han Chinese), offering relatively controlled population variability and ensuring study reliability due to the typically abundant periorbital fat in this group (29). Acquiring more comprehensive metrics may yield more accurate and nuanced results. CR features, renowned for their interpretability, are widely used in other medical fields (e.g., malignant tumor differentiation, disease prognosis) (30, 31). Their integration with ML methods achieved robust predictive efficacy in this context. Current research on periorbital fat assessment using ML combined with imaging remains exploratory, with limited reference study designs. Multiclass studies are particularly scarce owing to their complexity and high costs (32).

As a multi-class classification model (three-class), optimizing and enhancing model performance presented a considerable challenge. The final model demonstrated robust capability in evaluating periorbital fat, thereby providing valuable insights for future research. Nevertheless, several inherent limitations should be acknowledged: First, the retrospective design involved a limited sample size with unavoidable selection bias, making more granular age stratification beyond three groups unfeasible. Second, the absence of standardized facial photographic documentation restricted phenotypic correlation analysis. Third, the lack of multi-center imaging datasets precluded rigorous validation of the model’s generalization capability across diverse populations and equipment. Periorbital aging is a process of coordinated degradation involving “bone–muscle–ligament–fat.” Clarifying the aging characteristics of these tissues is a key objective in rejuvenation surgery. Within a limited timeframe, we aim to investigate changes in one specific tissue type rather than pursuing a comprehensive analysis of the overall aging process. Future efforts will focus on expanding datasets, refining feature engineering for periorbital fat and other anatomical substructures, and validating robustness through external cohorts. Ultimately, we aim to translate this model into a clinical decision-support tool integrated with electronic health records.

We attempted to investigate the periorbital skin, muscle, and fat as a unified composite (Supplementary Figures S3, S4); however, the performance of the developed CR, ML, CR + ML, and Stacking ensemble learning models failed to surpass that of the models based solely on the three fat compartments (Supplementary Figure S5; Supplementary Tables S1, S2). Among these, the MLP (CR + ML) model emerged as the best-performing combination, yielding a macro-average AUC of 0.771 (95% CI: 0.683–0.847) and an F1 score of 0.614. We attribute this to the fact that distinct anatomical regions may exhibit heterogeneous characteristics across different age groups; consequently, region-specific feature extraction contributes to enhanced model accuracy. Furthermore, given the high sensitivity of periorbital adipose tissue to aging, the utilization of multiple Regions of Interest (ROIs) for multimodal model development serves to improve the discriminative power of the models.

Artificial intelligence possesses the intrinsic capacity to capture latent, yet critical, feature information, thereby assisting in the resolution of significant clinical challenges. For instance, a study in the field of endocrinology demonstrated the feasibility of identifying prediabetic patients using solely a single-lead electrocardiogram (Lead I) (33). This approach achieved an area under the receiver operating characteristic curve (AUROC) of 0.844 (sensitivity: 0.823; specificity: 0.702) in an external validation cohort.

**Figure 5:**
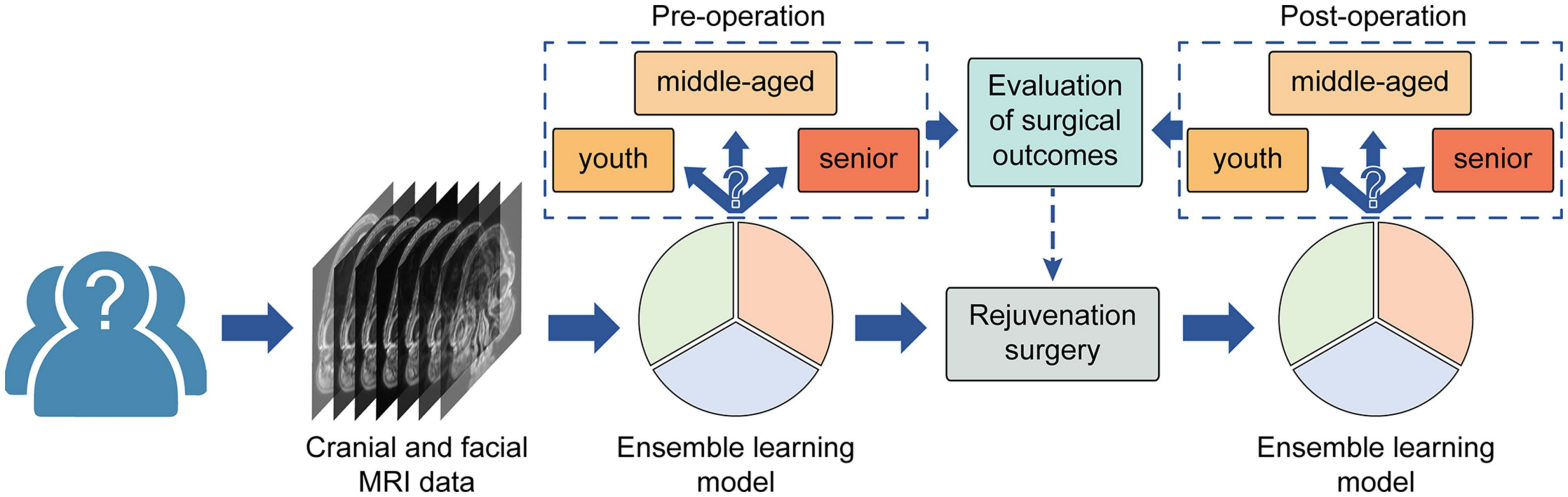
The application of the ensemble learning model in facial rejuvenation surgery.

Four types of models were developed sequentially: single-feature models, feature fusion models, and the ensemble learning model. The optimal ensemble learning model can assess the status of periorbital fat across the entire adult age spectrum, providing a radiological perspective on whether the periorbital fat status aligns with the normal status expected for the patient’s age group. If the evaluation indicated that a patient’s periorbital fat had prematurely advanced to the next age group, this finding suggested a higher necessity for the patient to undergo periorbital rejuvenation treatment. Moreover, the model holds promise as a reference for pre and post-operative evaluation of periorbital rejuvenation treatments (Figure 5). Improvement in the assessed age group based on periorbital fat status can reflect the efficacy of periorbital rejuvenation surgery.

## 5 Conclusion

The prediction models developed from both the CR features and ML features of periorbital fat successfully discriminated populations across three distinct age groups. Fusion CR and ML features enhanced the model’s capability to discriminatory capability between these age groups. Subsequently, the prediction probabilities generated by the CR-ML fusion model were utilized to construct a stacking ensemble learning model, which further improved the discriminatory accuracy across age strata. Continued refinement of training data and parameter optimization will provide clinicians with a straightforward and efficient tool to evaluate periorbital fat status. This model is anticipated to become a pivotal metric for assessing periorbital fat dynamics, thereby offering robust clinical support for periorbital rejuvenation surgeries.

## Data Availability

The raw data supporting the conclusions of this article will be made available by the authors, without undue reservation.
