# Supplementary material for: A multi-model fusion approach incorporating conventional radiological and machine learning features across age spectrum for periorbital fat status prediction
Source: Front Med (Lausanne). 2026 Feb 25;13:1752016. doi: 10.3389/fmed.2026.1752016 (PMC12978258; doi:10.3389/fmed.2026.1752016)
Supplement: Supplementary file 1 [file Data_Sheet_1.pdf]

Supplementary Material

1 Supplementary Data

1.1 Region of interest segmentation

Based on reviewing previous research data, three anatomical regions of periorbital fat were identified (Fig. 2A, Supplementary Figure 1, Supplementary Figure 2): retro-orbicularis oculi fat (ROOF), situated between the inferior frontal vestibule (superior boundary) and the orbicularis retaining ligament (inferior boundary), lateral to the supraorbital neurovascular bundle emerging from the supraorbital foramen (medial boundary), and medial to the lateral orbital rim (lateral boundary), posterior to the orbicularis oculi muscle; sub-orbicularis oculi fat (SOOF), located between the zygomatico-cutaneous ligament (superior boundary) and the orbicularis retaining ligament (inferior boundary), lateral to the angular vein (medial boundary), posterior to the orbicularis oculi muscle and the lateral orbicularis thickening (LOT); and deep medial cheek fat (DMCF), positioned between the orbicularis retaining ligament (ORL; superior boundary) and the maxillary ligament (MxL; inferior boundary), lateral to the pyriform ligament (medial boundary), medial to the zygomaticus major muscle and the buccal fat pad membrane (lateral boundary), posterior to the levator labii superioris, levator labii superioris alaeque nasi, and orbicularis oculi muscles.

1.2 Statistical analysis

All data analyses were conducted using open-source libraries in Python, including pandas (v2.3.1), NumPy (v2.0.2), SciPy (v1.13.1), scikit-learn (v1.6.1), Matplotlib (v3.9.4), Seaborn (v0.13.2), and Pingouin (v0.5.5) for statistical and visualisation purposes.

1.3 Comprehensive study of various tissues in the periorbital area

We have annotated the skin, muscle, SOOF (sub-orbicularis oculi fat), ROOF (retro-orbicularis oculi fat), and DMCF (deep medial cheek fat) as a single, unified ROI (Supplementary Figure 3). By expanding the study area and treating it as a holistic ROI, we have performed a more comprehensive extraction of both Conventional Radiomics (CR) and Machine Learning (ML) features.

1.3.1 Workflow for Feature Extraction and Model Development

Consistent with the methodology established in this study, we performed data processing and model evaluation (Supplementary Figure 4).

1.3.2 Development of CR, ML, and CR+ML Models

We compared the test set evaluation metrics for models developed using a single unified ROI versus those using the original three ROIs (Supplementary Table 1). The results indicate that while the single-ROI approach improved the primary performance metrics for certain models—specifically the CR-based GBM model, as well as the CR+ML-based MLP and SVM models—none of these improvements

surpassed the performance of the original optimal models. The best-performing model for each feature category remains the one developed based on the three-ROI segmentation.

1.3.3 Development of Stacking Ensemble Learning Models

We selected the top five model combinations for a comparative analysis of their performance metrics (Supplementary Table 2). It is evident that all ensemble models developed based on the single ROI exhibited suboptimal performance. The highest-performing base model combination consisted of MLP, MLR, NN, and RF, which achieved an AUC-macro (95% CI) of 0.682 (0.591–0.771) and an F1-score of 0.286. These metrics fall short of the requirements for a robust and reliable predictive model, with their overall performance being inferior to that of most individual base models.

1.3.4 Comparison of Other Performance Metrics for the Optimal Model

Through visual analysis of model performance, it is evident that both the ROC curves and confusion matrices for the model developed using a single ROI are inferior to those of the model developed using the three original ROIs (Supplementary Figure 5).

2 Supplementary Figures and Tables

2.1 Supplementary Figures

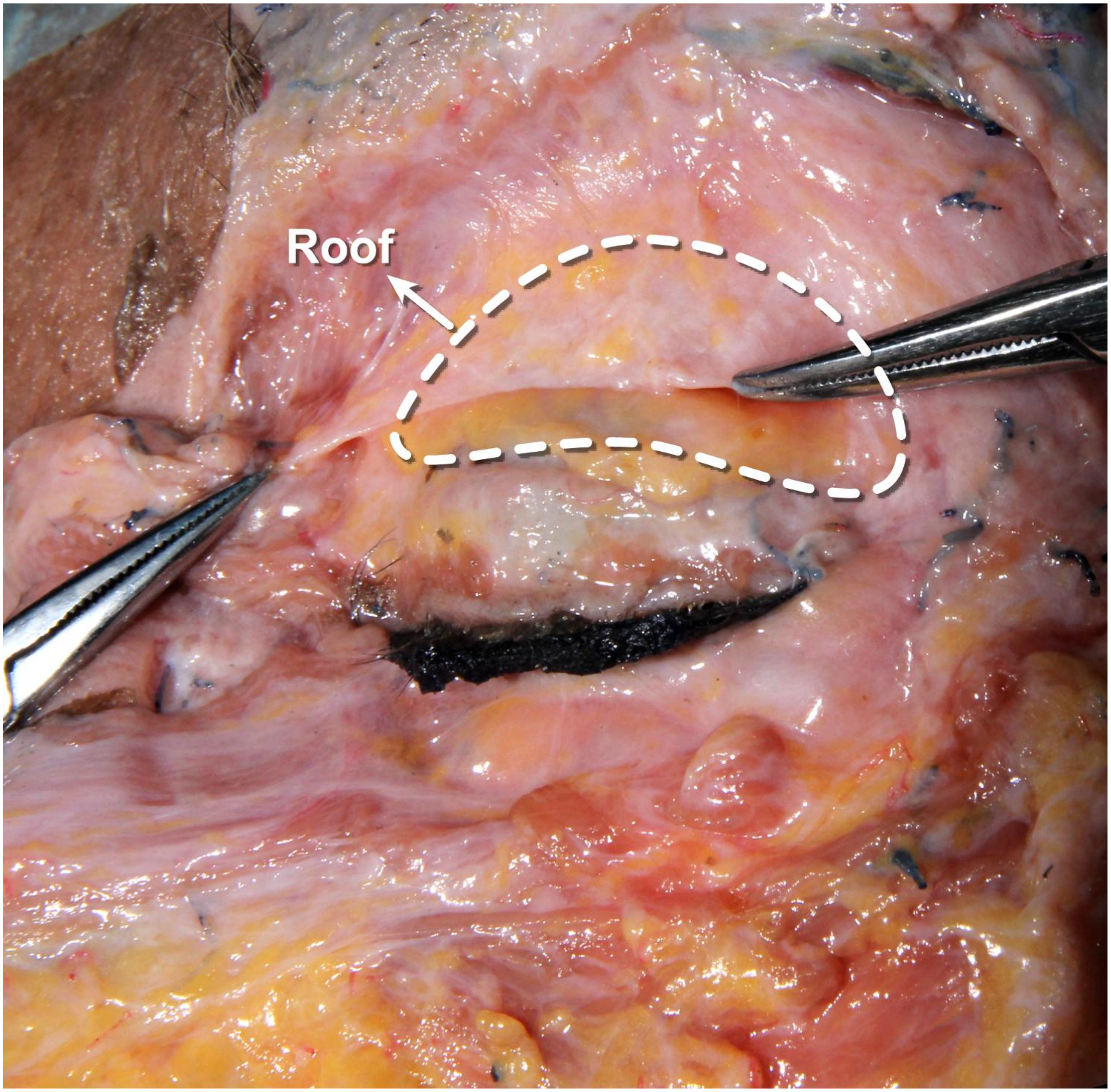

Supplementary Figure 1. Retro-orbicularis oculi fat (ROOF) at the anatomical position of the fresh corpse head.

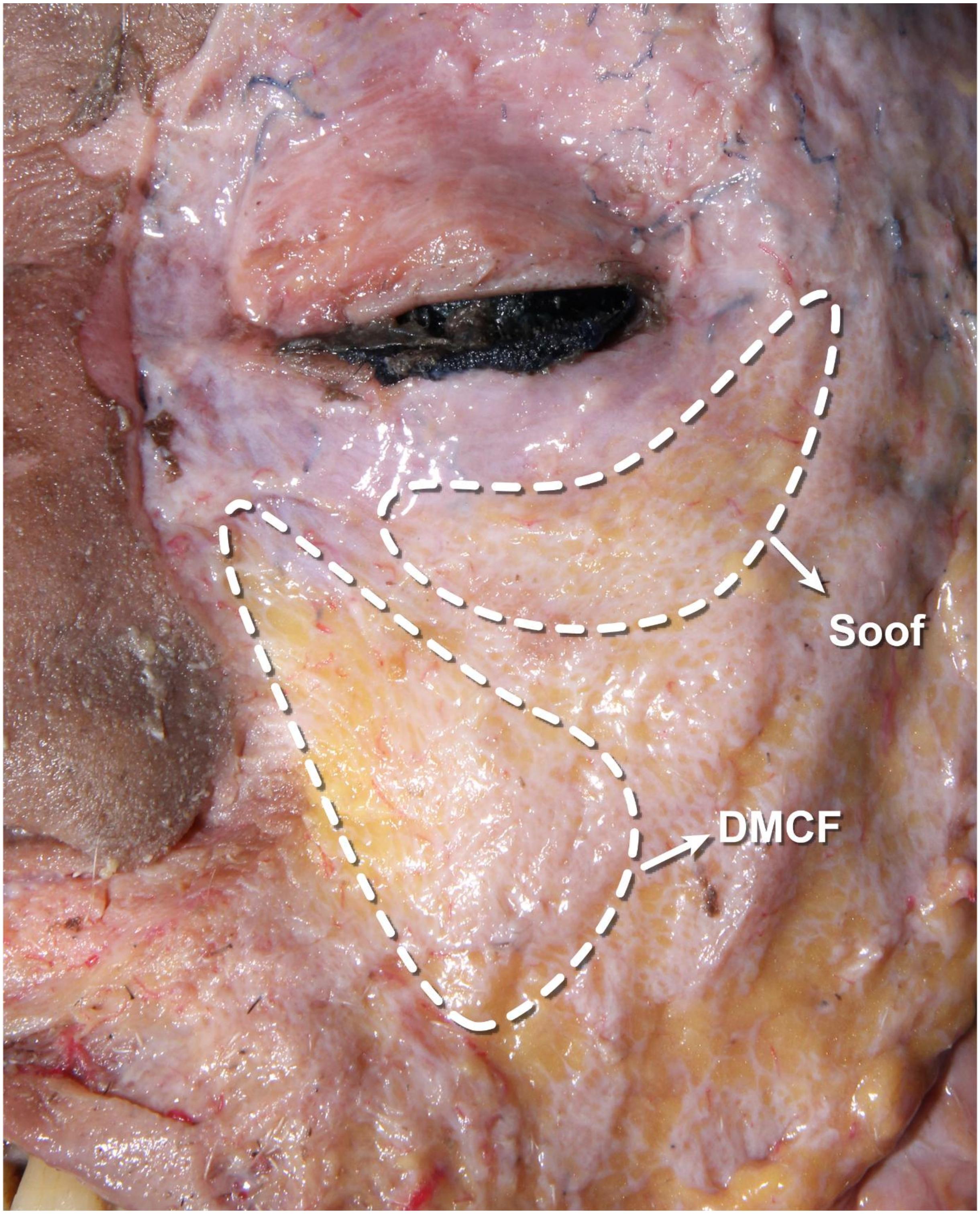

Supplementary Figure 2. Sub-orbicularis oculi fat (SOOF) and deep medial cheek fat (DMCF) at the anatomical position of the fresh corpse head.

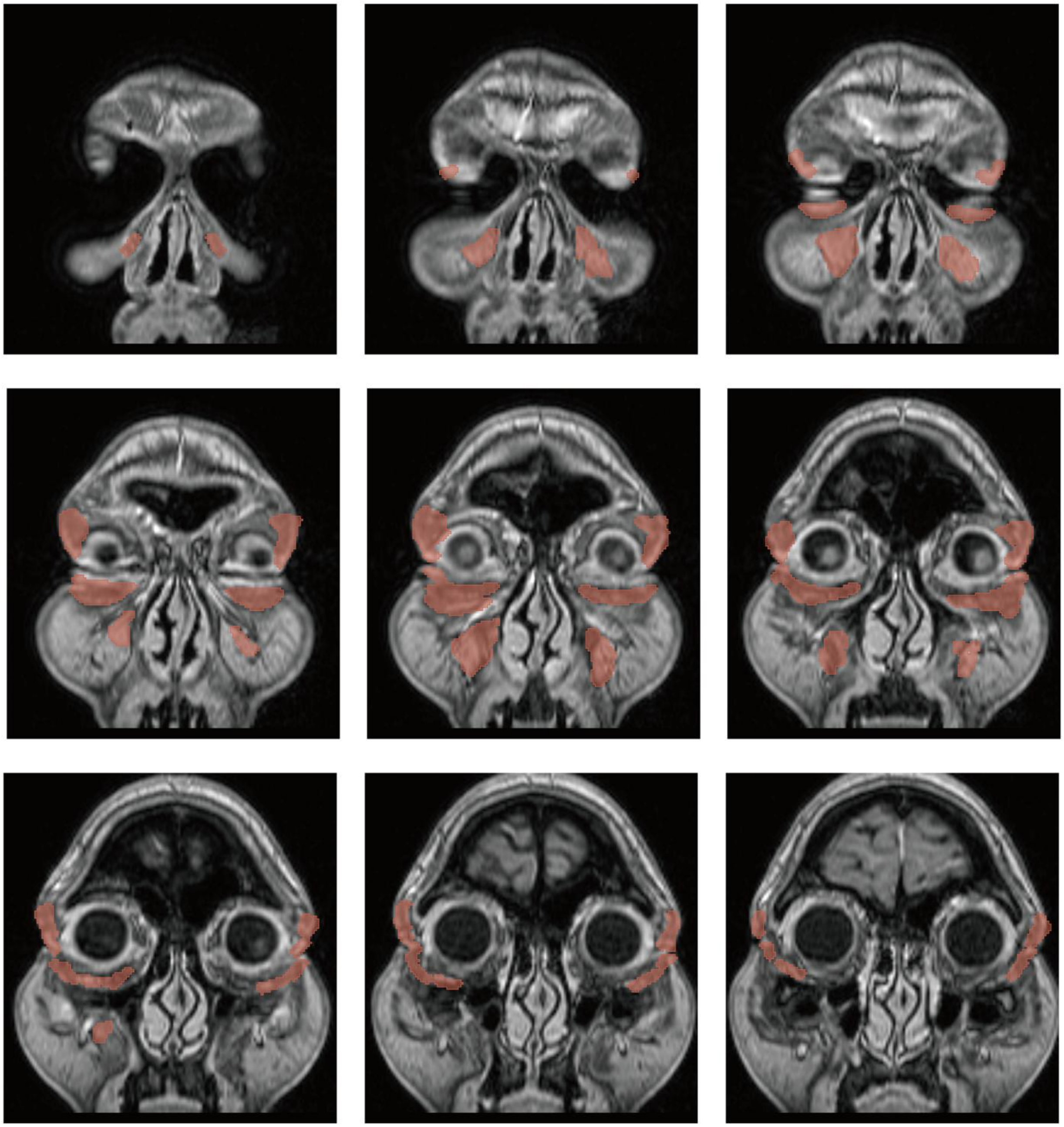

Supplementary Figure 3. Expand the annotation range: Mark the skin, muscles, SOOF (sub-orbicularis oculi fat), ROOF (retro-orbicularis oculi fat), and DMCF (deep medial cheek fat) as a single integrated ROI.

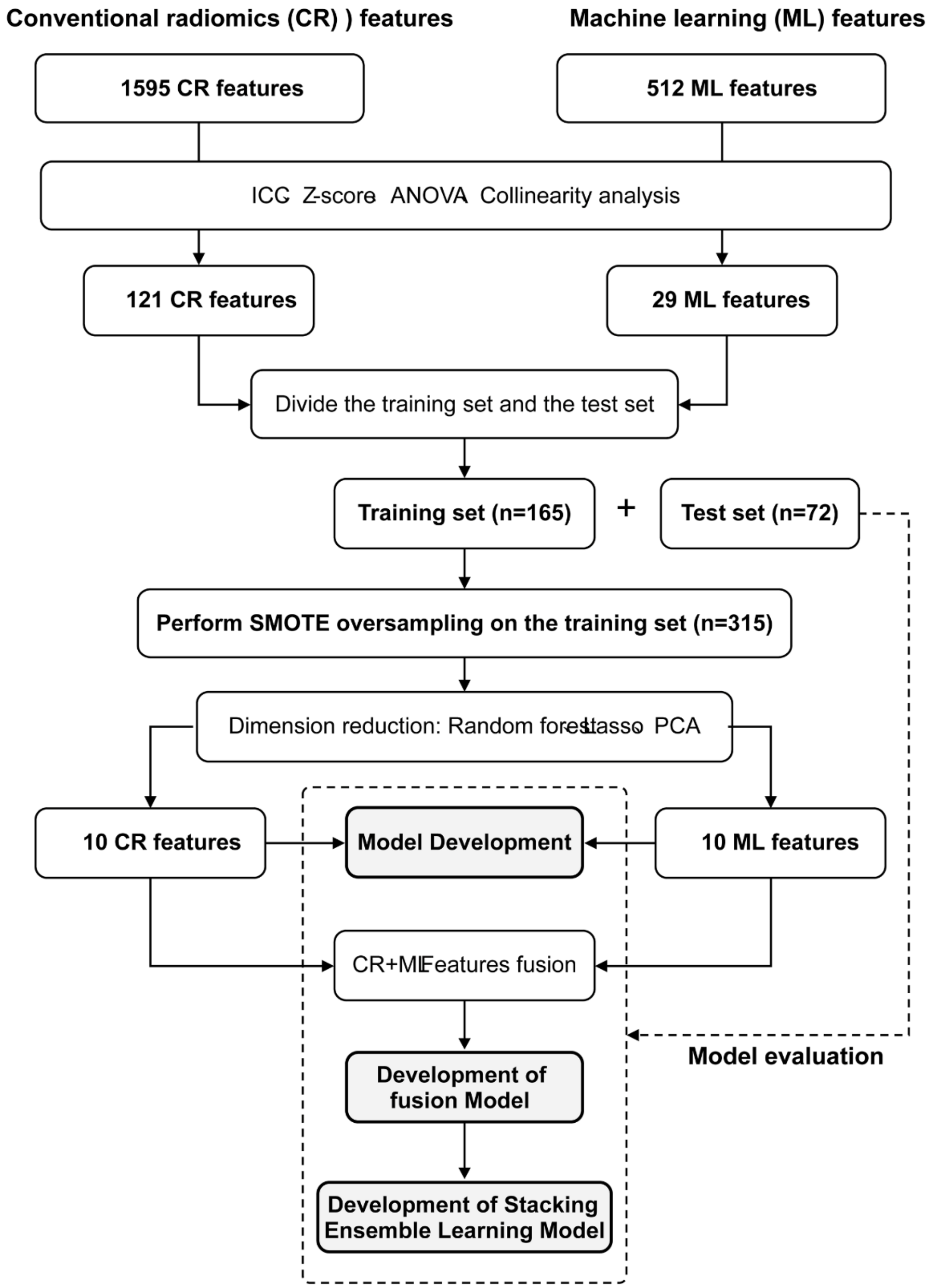

Supplementary Figure 4. Data processing and model development based on 1 ROI.

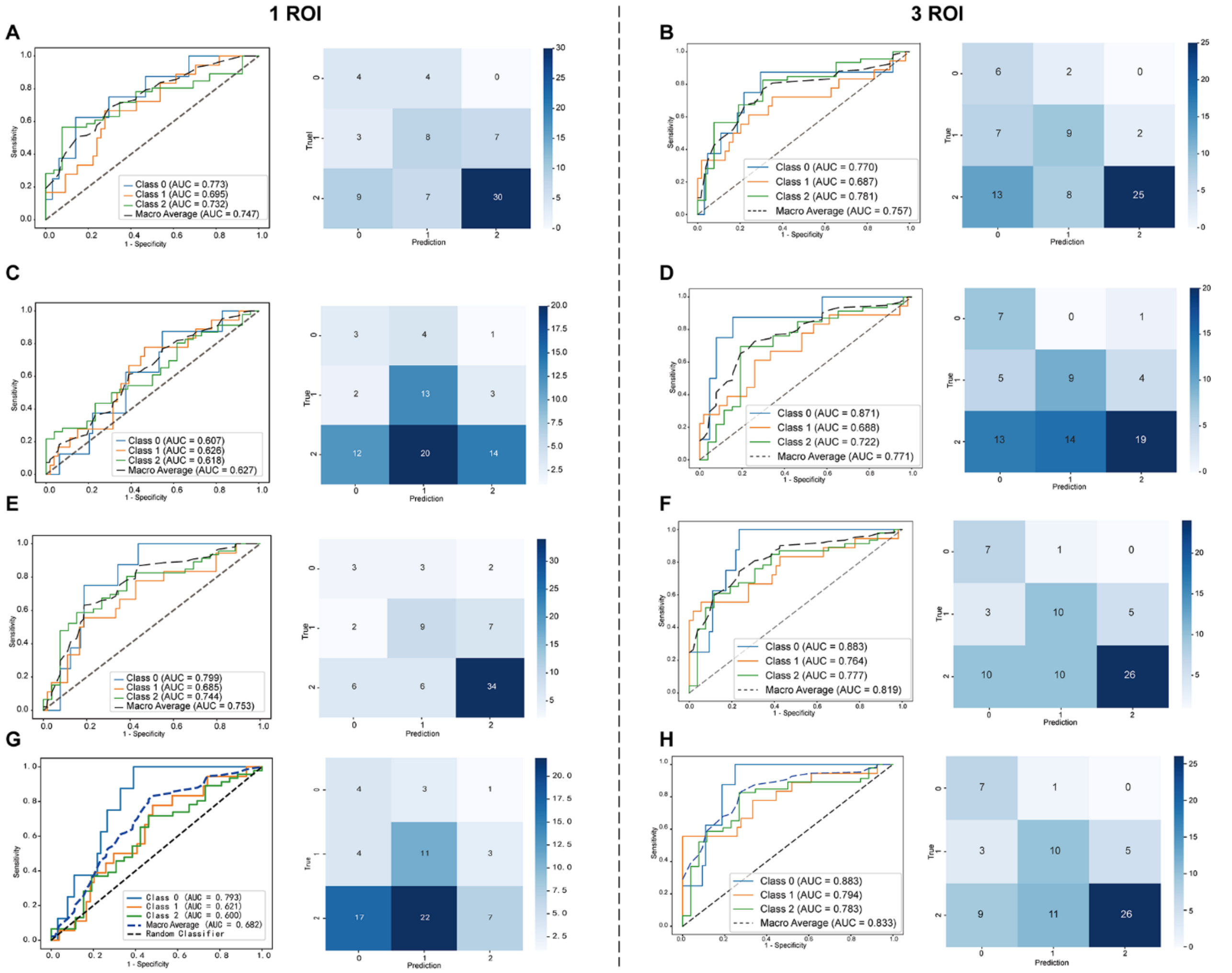

Supplementary Figure 5. Comparison of performance metrics on the test set for the optimal models. The left and right panels present the visual analysis of optimal models developed using a single ROI and three ROIs, respectively. (A) CR-based NN model (single ROI); (B) CR-based Naive Bayes model (three ROIs). (C) ML-based XGBoost model (single ROI); (D) ML-based MLR and Naive Bayes model (three ROIs). (E) Fusion model based on CR+ML features (SVM, single ROI); (F) Fusion model based on CR+ML features (MLR, three ROIs). (G) Stacking ensemble model based on base models (MLP, MLR, NN, RF). (H) Stacking ensemble model based on base models (GBM, LGBM, NN).

2.2 Supplementary Tables

Supplementary Table 1. Performance comparison between models developed using a single ROI and those using three ROIs. AUC-macro, AUC macro-average; AUC, the area under the receiver operating characteristic curves; Acc, accuracy; PPV, positive predictive value; Sen, sensitivity; F1, F1-score; CI, confidence interval; CR, conventional radiation; ML, machine learning; MLR, multiclass logistic regression; MLP, multilayer perceptron; NN, convolutional neural network; RF, random forest; GBM, gradient boosting machine; XGBoost, extreme gradient boosting; LGBM, light

gradient boosting machine. *Sen is calculated in multi-class problems using a weighted average, which is mathematically equal to Acc.

	1 ROI				3 ROI			
	Acc (Sen)*	PPV	F1	AUC-macro	Acc (Sen)*	PPV	F1	AUC-macro
**GBM**								
CR	0.514	0.574	0.532	0.708(0.597-0.795)	0.472	0.633	0.508	0.706 (0.583-0.796)
ML	0.431	0.536	0.461	0.540(0.408-0.670)	0.361	0.502	0.391	0.682 (0.573-0.773)
CR+ML	0.444	0.508	0.470	0.635(0.536-0.728)	0.5	0.626	0.53	0.716 (0.599-0.816)
**LGBM**								
CR	0.486	0.561	0.514	0.623(0.502-0.724)	0.472	0.669	0.51	0.715 (0.603-0.806)
ML	0.375	0.503	0.392	0.547(0.442-0.651)	0.403	0.554	0.412	0.676 (0.568-0.773)
CR+ML	0.556	0.615	0.575	0.672(0.564-0.770)	0.528	0.667	0.561	0.767 (0.668-0.85)
**MLP**								
CR	0.500	0.619	0.538	0.694(0.565-0.796)	0.528	0.73	0.579	0.737 (0.634-0.82)
ML	0.444	0.609	0.482	0.631(0.511-0.753)	0.458	0.589	0.477	0.682 (0.565-0.793)
CR+ML	0.569	0.724	0.614	0.722(0.623-0.808)	0.542	0.646	0.577	0.713 (0.59-0.818)
**MLR**								
CR	0.569	0.691	0.606	0.744(0.638-0.831)	0.583	0.712	0.62	0.748 (0.623-0.843)
ML	0.431	0.614	0.442	0.611(0.485-0.718)	0.486	0.635	0.504	0.771 (0.651-0.862)
CR+ML	0.583	0.676	0.614	0.771(0.683-0.847)	0.597	0.721	0.619	0.806 (0.703-0.884)
**Naive Bayes**								
CR	0.514	0.612	0.541	0.689(0.585-0.787)	0.556	0.736	0.598	0.757 (0.628-0.856)
ML	0.347	0.573	0.370	0.566(0.435-0.687)	0.361	0.501	0.365	0.624 (0.506-0.733)
CR+ML	0.444	0.603	0.495	0.621(0.486-0.748)	0.542	0.732	0.558	0.775 (0.665-0.856)
**NN**								
CR	0.583	0.651	0.607	0.747(0.640-0.838)	0.583	0.7	0.614	0.749 (0.639-0.837)
ML	0.403	0.578	0.426	0.579(0.463-0.695)	0.458	0.666	0.476	0.706 (0.593-0.804)
CR+ML	0.583	0.662	0.603	0.739(0.634-0.833)	0.597	0.694	0.615	0.819 (0.720-0.893)
**RF**								
CR	0.486	0.580	0.516	0.678(0.569-0.775)	0.528	0.709	0.57	0.727 (0.605-0.822)
ML	0.444	0.533	0.463	0.567(0.444-0.678)	0.417	0.586	0.422	0.717 (0.619-0.800)
CR+ML	0.583	0.627	0.601	0.708(0.614-0.799)	0.597	0.725	0.612	0.779 (0.691-0.851)
**SVM**								
CR	0.514	0.507	0.510	0.693(0.581-0.792)	0.569	0.652	0.594	0.741 (0.648-0.825)
ML	0.486	0.532	0.503	0.526(0.401-0.648)	0.5	0.592	0.522	0.727 (0.616-0.817)
CR+ML	0.639	0.660	0.648	0.753(0.661-0.838)	0.556	0.634	0.579	0.739 (0.637-0.829)
**XGboost**								
CR	0.486	0.557	0.515	0.620(0.497-0.728)	0.458	0.642	0.496	0.748 (0.651-0.824)
ML	0.417	0.604	0.424	0.627(0.501-0.717)	0.417	0.604	0.419	0.721 (0.614-0.807)
CR+ML	0.597	0.646	0.614	0.698(0.587-0.793)	0.528	0.689	0.548	0.789 (0.692-0.861)

Supplementary Table 2. AUC-macro, AUC macro-average; AUC, the area under the receiver operating characteristic curves; Acc, accuracy; PPV, positive predictive value; Sen, sensitivity; F1, F1-score; CI, confidence interval; CR, conventional radiation; ML, machine learning; MLR, multiclass logistic regression; MLP, multilayer perceptron; NN, convolutional neural network; RF, random forest; GBM, gradient boosting machine; XGBoost, extreme gradient boosting; LGBM, light

gradient boosting machine. *Sen is calculated in multi-class problems using a weighted average, which is mathematically equal to Acc.

	1 ROI					3 ROI			
Model	Acc (Sen)*	PPV	F1	AUC-macro (95% CI)	Model	Acc (Sen)*	PPV	F1	AUC-macro (95% CI)
MLP, MLR, NN, RF	0.306	0.501	0.286	0.682 (0.591-0.771)	GMB, LGBM, NN	0.597	0.69	0.614	0.833 (0.737-0.902)
MLP, MLR, NN, RF, XGboost	0.306	0.501	0.286	0.682 (0.576-0.768)	LGBM, NN	0.597	0.69	0.614	0.833 (0.733-0.904)
LGBM, MLP, MLR, NN, XGboost	0.319	0.527	0.279	0.678 (0.582-0.766)	LGBM, MLP, NN, Naive bayes	0.597	0.713	0.617	0.827 (0.728-0.900)
GBM, MLP, MLR, NN, XGboost	0.319	0.528	0.280	0.675 (0.581-0.767)	GMB, LGBM, MLP, NN, Naive bayes	0.597	0.713	0.617	0.826 (0.728-0.903)
GBM, LGBM, MLP, MLR, NN, XGboost	0.319	0.527	0.279	0.670 (0.567-0.754)	GMB, LGBM, MLP, NN, Naive bayes, XGBoost	0.597	0.713	0.617	0.826 (0.725-0.899)
